# A peer interview qualitative study exploring support for carers of people with comorbid autism and eating disorders

**DOI:** 10.1186/s40337-021-00397-6

**Published:** 2021-03-31

**Authors:** Emma Kinnaird, Madeleine Oakley, Vanessa Lawrence, Sukhi Shergill, Kate Tchanturia

**Affiliations:** 1 King’s College London, Department of Psychological Medicine, Institute of Psychiatry, Psychology and Neuroscience, London, UK; 2 King’s College London, Health Services and Population Studies Department, Institute of Psychiatry, Psychology and Neuroscience, London, UK; 3 King’s College London, Psychosis Studies, Institute of Psychiatry, Psychology and Neuroscience, London, UK; 4 National Eating Disorder Service, South London and Maudsley NHS Foundation Trust, London, UK

**Keywords:** Carers, Qualitative study, Psychological support, Innovation, Autism, Eating disorders, Peer interviewing

## Abstract

**Background:**

Carers of people with eating disorders (EDs) are known to experience a lack of support, high levels of unmet needs and resulting distress. Specific support and interventions for carers may benefit both the carer, and their loved one with an ED. Individuals with co-occurring autism and EDs may present with additional needs and difficulties relating to their Autism Spectrum Condition (ASC) that impact their carers. However, there is a lack of research exploring whether carers of people with ASC and EDs have specific support needs, and what kinds of support may be most beneficial for this population.

**Methods:**

This study used a qualitative interview design, utilising peer interviews. Eleven carers participated in interviews about their experiences as a carer, and their views on existing support systems and potential improvements. As the study took place during the initial UK coronavirus lockdown, the impact of the lockdown also emerged as a topic during the interviews. Interviews were transcribed and analysed using thematic analysis.

**Results:**

Five themes were identified: challenges associated with co-occurring Autism and EDs, a lack of existing support for carers from healthcare services, the personal impact of caring for someone with both ASC and EDs, ideas for how carers can be best supported, and the impact of the coronavirus on carers.

**Conclusions:**

Carers of loved ones with both ASC and EDs described the experience as having a significant personal impact on their lives, but also experienced a lack of support from healthcare services. There was a perception that caring for someone with both an ASC and EDs presents additional challenges compared to caring for someone with an ED only, and that this population therefore requires specialised support. Recommendations for possible support options, and for further research, are outlined.

## Plain English summary

Caring for someone with an eating disorder can be challenging, and come at great personal cost. We know that individuals with both an eating disorder and autism can experience further difficulties compared to someone with an eating disorder only, which might present an additional challenge for their carer. In this study, we wanted to explore the experiences of carers of people with both autism and an eating disorder, to understand how healthcare services can best support this population. We found that these carers felt frustrated and isolated. They described not receiving appropriate support from healthcare services, including services not understanding how to adapt treatments for someone with autism and an eating disorder. This led to carers taking a greater role in managing their loved one’s illness and recovery, forcing them to make personal sacrifices such as giving up their job. We outline possible ways healthcare services can best support these carers.

## Background

Eating disorders (EDs) are characterised by disturbed eating, food consumption, and related behaviour that significantly impacts physical and psychosocial health [1]. Typical ED onset is in adolescence/ early adulthood, at a time when people with EDs may still be living at home and reliant on family support [2]. As ED treatments typically take place on an outpatient basis, parents may also take on the role of helping implement and ensure treatment compliance whilst their child is at home. For example, family-based approaches explicitly encourage and help parents to support their child’s treatment, particularly around mealtimes and nutrition [3, 4].

Therefore, a young person developing an ED will have a profound impact on their parents, who often find themselves acting as carers for their child and playing a key role in their treatment. Carers of people of EDs consistently report high levels of psychological distress and perceived burden, which appear to be driven by unmet practical and emotional support needs [5–7]. Caring for someone with an ED may also come at a great financial cost for families, including leading carers to reduce working hours to support their child [8]. The pressures associated with this experience can lead to the carers developing unhelpful coping strategies, including accommodating or enabling ED behaviours and having high levels of expressed emotion (emotional overinvolvement, hostility, over-protectiveness, or controlling behaviour), which may contribute to poorer family functioning and help maintain the ED itself [5, 9–11]. In recognition of the key role that carers play in ED treatment and recovery, particularly for younger people, NICE guidelines for ED services in the UK specify that clinicians should assess carer needs and offer support and psychoeducation as appropriate [12]. In line with this, a number of interventions designed to support carers and families of people with EDs have been developed [13]. Psychoeducational and skills training-based interventions have been found to benefit carers and their loved ones with an ED, reducing carer distress, perceived burden, and levels of expressed emotion [14–16].

Whilst there is now a significant body of literature detailing the experiences of carers for people with EDs, and potentially beneficial interventions, there is much less research on the support needs of parents who care for someone with diagnoses of both an ED and Autism Spectrum Condition (ASC) Autism (we use this terms since it was established that people with autism don’t like using the term Autism spectrum disorders). ASC is a neurodevelopmental condition associated with persistent difficulties in social interaction and social communication, and restrictive and repetitive patterns of behaviour, interests, or activities [1]. Research suggests that autistic traits are heightened in ED populations, particularly in those with anorexia nervosa [17, 18]. Gold-standard observational diagnostic measures combined with developmental assessments suggest that autism prevalence in AN may be around 10%, compared to a general population prevalence of ~ 1% [19, 20]. Individuals with both an ED and a diagnosis of ASC may experience additional issues compared to their neurotypical peers, including sensory problems motivating food restriction, difficulties engaging in treatment due to communication differences, and high levels of cognitive rigidity making it difficult to change ED-related behaviours and cognitions [21, 22]. Researchers measured parenting stress in a sample of 298 children with ASD aged 3–14 years and found that parents of children with serious behavioural problems reported the highest degrees of stress [23]. Other studies had demonstrated that ASD children with disruptive behaviour created high levels of stress in parents [24–26]. Previous researchers [27] also found that disruptive behaviours in children with ASD caused parental stress from the early years through to adulthood. Problem behaviours in children with ASD include temper tantrums, self-harm, aggression to the self and others and self-stimulation, [28].

The addition of an autism diagnosis may also present challenges to carers of people with EDs. Similarly to AN, parents of children with ASC experience high levels of perceived burden and distress relating to their caregiving role that are closely linked to unmet needs [29, 30]. Research suggests that parents of children with ASC may experience a lower quality of life compared to parents of children with mental health problems [31]. This likely reflects the nature of ASC: unlike mental health problems, autism is a neurodevelopmental condition that will be present across the individual’s lifetime, and is associated with continuing difficulties with daily living into adulthood [26]. In contrast to EDs, support and interventions for people with ASC and their caregivers are not typically based on the possibility of recovery: rather, they may be more focused on improving or adapting around autism spectrum-associated difficulties. These can include supporting communication, skills-based training for carers, and improving carer synchrony with their child [32].

Therefore, children with autism are likely to have specific long-term support needs across their lifespan, and may remain dependent on their families for care [29, 33]. Consequently, parents caring for a child with ASC are likely to experience additional challenges that may not be typically experienced by a parent caring for a child with an ED only, and so may not be addressed by existing caregiver interventions or support for ED caregivers. To date, two qualitative studies have explored the experiences of carers of children with a diagnosis of both ASC and AN [21, 34]. Brede et al. (2020) explored the relationship between restrictive eating difficulties and ASC with carers, patients and clinicians. They found that ASC and AN are closely intertwined, and that women with both conditions have different needs compared to people with AN only. Adamson et al. (2020) also explored this area, focusing on the perspective of interviewing carers about treatment adaptations for people with both ASC and AN. This study similarly found that ASC and AN were highly interrelated and associated with unique treatment needs compared to people with AN only, such as requiring support around sensory difficulties and longer time in treatment. Although Adamson et al. (2020) focused on carer views on treatment adaptations for their loved ones, the findings indicated that the carers themselves experience high levels of unmet needs, a perceived lack of support from existing services, and subsequent feelings of isolation and frustration. This study suggested that this group may potentially benefit from support designed specifically for carers of people with autism and AN.

Therefore, the primary aim of this study was to use qualitative interviews to further explore the support needs of carers of loved ones with co-occurring ASC and AN, and to investigate in more detail carer views on how this population can best be supported. In the context of previous findings indicating a perceived lack of support and alienation from existing ED services, the existing study used a peer interviewing approach, in which the interviewer (shared first author MO) was also a carer of a young person with autism [35]. Peer interviewing is becoming an increasingly popular approach in qualitative research: it has the benefit of increasing engagement and comfort of participants who may have previously had difficult relationships with clinical services, as previously described by this population [35, 36].

Whilst the initial aim of the study was to explore the support needs of carers of loved ones with co-occurring ASC and AN, shortly before data collection commenced in March 2020 the UK entered lockdown due to the coronavirus pandemic. This had the result of some ED treatment services experiencing a period of disruption, including inpatient services closing and outpatient treatments moving online. As this had a significant impact on the carers interviewed in this study, the analysis includes reflections on carer experiences during the pandemic.

## Methods

This study used a qualitative design, carrying out semi-structured peer interviews with carers of people with a diagnosis of an ED and autism. All participants in the interview studies gave written informed consent, and ethical approval was obtained from London-City and East Research Ethics Committee and South London (18/LO/0050).

### Recruitment

A total of 11 carers participated in the interviews. Four carers were recruited as their child was receiving support from the PEACE pathway (Pathway for Eating disorders and Autism developed from Clinical Experience) at the South London and Maudsley (SLAM) National Health Service (NHS) Trust National ED service. The PEACE pathway is a novel specialist ED treatment pathway for individuals with diagnosed or suspected autism [31]. An additional seven carers were recruited through advertising on Twitter using the PEACE pathway Twitter account (@PEACE_Pathway). Participants were considered eligible if they had current caring responsibilities for someone with diagnoses of both an ED and ASC. For participants recruited from the PEACE pathway, diagnoses were confirmed from clinical notes. One participant’s child did not yet have an official ASC diagnosis, but had scored positively on a gold-standard diagnostic assessment (the Autism Diagnostic Observational Schedule). For participants recruited on social media, diagnoses were self-reported by the carers (typically obtained from autism specialist clinical services) only and not further validated by our research team. All authors read interview transcripts and regularly met to discuss the data, until it was felt that data saturation had been reached. Data saturation was defined as the point at which no new ideas or information were being raised by the interviews.

### Data collection

Due to the COVID-19 pandemic, all interviews were carried out virtually by MO between March and July 2020. MO is a carer for a young person with ASC, as well as a researcher and family therapist.

The interviews were initiated using a pre-developed interview schedule, based on previous research [34, 37] that explored carers’ needs by the present research team. The questions were as follows:

- *When did your son or daughter receive their diagnosis of autism?*
- *What treatment is he/she receiving?*
- *Did he/she have any subsequent assessments?*
- *Is there anything you’d like to say about your experience of getting the assessments or treatments?*
- *If you could create the treatment your son/daughter needs, what would it look like?*
- *Have you got any thoughts about what help should be offered to people in your position, family carers?*
- *What has been the impact of this on you as a carer?*
- *What does improvement look like? For your son/daughter? For you? For the family?*

The above schedule was then revised iteratively over the course of the interviews to follow the concerns of participants. The Interviews were conducted over the telephone, or using Microsoft Teams or Zoom software, according to the carer’s preference, and lasted 30–40 min (mean interview length 35 min). As found elsewhere, the multiple contacts involved in setting up these appointments and resolving any technical difficulties assisting in establishing rapport with participants. All interviews were recorded and transcribed by MO. Data collection and analysis were conducted in parallel and recruitment ceased when we reached inductive theoretical saturation, the point at which no new themes or sub-themes were emerging from the data. In an inductive approach, the themes are all found in the data itself. Saunders et al. (2018) have the following definition “inductive theoretical saturation focuses on the identification of new codes or themes, and is based on the number of such codes or themes rather than the completeness of existing theoretical categories. This can be termed inductive thematic saturation. In this model, saturation appears confined to the level of analysis; its implication for data collection is at best implicit.” [38].

### Participant characteristics

A total of 11 people participated in the interviews. All were parents of a child or adult with autism and an ED: three were fathers, and eight were mothers. Three participants were full time carers for their child, six were employed, and two were retired. Carer ages ranged from 38 to 74 years, with a mean age of 55 years. The people cared for by the participant comprised three males and eight females. Their ages ranged from 12 to 36 years, with a mean age of 23 years. According to parent reports, all patients had been diagnosed with ASC (mean diagnosis age 16.63 years), were verbal, and diagnosed with an ED (mean diagnosis age 13.13 years). Nearly all patients were diagnosed with restrictive AN (*n* = 9): One patient had a diagnosis of binge-purge AN, and One patient had received ED treatment due to binge eating disorder (BMI in obesity range). All patients had additional mental health difficulties: all parents reported their child experiencing anxiety symptoms, *n* = 7 had been diagnosed with obsessive-compulsive disorder, *n* = 4 had a diagnosis of borderline personality disorder, and *n* = 2 had a diagnosis of body dysmorphic disorder. One patient was reported to have a comorbid diagnosis of pathological demand avoidance (this is behavioural descriptor not in the current DSM or ICD diagnoses). Carers had been involved in the following different therapeutic approaches – a 6 month course on Body Dysmorphic Disorder (Carers 1&2); Family Therapy (Carers 3& 6); Family-based Therapy (Carers 4 & 6); Cognitive Remediation Therapy (Carers 5 & 10); Multi-family Therapy (Carer 6); Counselling (Carer 7); OT (Carer 7); Specialist Autism College (Carer 7); Multi-agency Behaviour Support Service (Carer 8); school-based Emotional Literacy Support Assistant (Carer 8); Psychiatrist (Carer 9); Specialist Autism School (Carer 10); Specialist OCD treatment (Carer 11).

### Analysis

Data was analysed using thematic analysis [39]. M.O., E.K. and K.T. read and re-read the transcripts in order to familiarise themselves with the data, and to increase sensitivity to key themes across the data set. In the second phase, these authors met to discuss the coding framework. The codes were based on the perceived relevance of the data to the authors’ pre-existing research aims: the researchers were following a structured set of questions which had been developed in a previous research study (Adamson et al. (2020) based on pre-existing ideas about the issues which might be of relevance to carers of family members with EDs and autism.

Although participants were not directly asked about the pandemic, seven out of the 11 participants (*n* = 7, 63.6%) discussed the impact of lockdown during the interviews. Therefore, specific codes were developed to reflect this topic. First author M.O. then applied these codes to the data, and collated data for each code. In the third phase, all authors reviewed the collated coding data and met to evaluate possible themes, which helped raise awareness to alternative interpretations of the data. Explicit and semantic themes were identified as the participants were interviewed using specific questions. Theme identification was also based on the perceived relevance to the research aims. Themes were then subsequently reviewed by authors and defined. Following this process, the following five themes were identified: Challenges associated with co-occurring autism and EDs; Lack of existing support; Impact on carers; Supporting the supporters; Coronavirus.

## Results

### Theme 1: challenges associated with co-occurring autism and EDs

All of the participants highlighted the additional challenges that this particular comorbidity brought to caring for their loved ones. Nearly all carers described difficulties getting an autism diagnosis for their loved one, or not recognising their child’s autism spectrum features due to an overshadowing mental health problem: all carers (*n* = 11) recalled that their loved one only received an autism diagnosis after receiving treatment for mental health problems, most commonly AN. There was a perception that delayed recognition of ASC resulted in later problems due to a lack of appropriate early support and interventions:

“It was very important, the diagnosis is, and I really honestly think if we had had the right intervention, and if, say, she’d been picked up at 8 years old, I really don’t think we’d have the problems we have today, because we would have put the right support in place, education-wise.” (Participant 10).

However, even once their child had been diagnosed with ASC, this did not necessarily result in appropriate care. Carers felt that some ED services struggled, or refused, to treat people with comorbid ASC:

“They have been helpful (clinical team) with the eating disorder, but because they don’t recognise the diagnosed pathological demand avoidance a lot of the advice they give are things that I don’t feel are suitable for [x name], because they don't account for some of the complexities.” (Participant 8).

Existing ED treatments were viewed as requiring adaptation as they felt that their loved ones presented with specific needs compared to those with EDs only. For example, carers described how their offspring presented with challenging behaviours, including extreme weight loss, suicidality, self-harm, and violence towards the carer themselves. There was a perception that the combination of the ASC and ED resulted in a more extreme presentation: one carer described how the comorbidity acted as a “full-on tsunami”:

“[The community treatment team] kept talking about “riding the wave of anorexia” but it wasn’t a wave, it was a full-on tsunami. [x- name] inability to regulate - we knew anyway that she probably did have autism, but that was diagnosed in hospital … [the community team] have no idea what this is like to live with, on a daily basis when somebody is in that starved state and has ASC.” (Participant 6).

As well as more extreme presentations, carers also described how their loved one’s ASC was associated with additional needs compared to EDs only, resulting in difficulties that were not always addressed by ED treatment. Perceived additional difficulties included sensory problems acting as a barrier to their child engaging in nutritional programmes, and a lack of insight into the ED itself. For example, the same carer described how her child’s difficulties with abstract thought made engaging in family therapy challenging:

“We could see that it was very different for [x name], she wasn’t able to access that ability to look at the future or anything like that, all the techniques that they were trying to use in the MFT - for example, we had to map out what she looked like/are like now, visually, what it was like with anorexia, what you wanted your future to look like and it was full of pictures and drawings and things like that. It made no difference; she ripped it up.” (Participant 6).

### Theme 2: lack of existing support

Despite the additional difficulties associated with caring for someone with both autism and an ED, carers reported experiencing a lack of help or support from services. This appeared to take two main forms: firstly, a perception that their loved one was not receiving support or treatment that considered the complexities associated with their autism. In particular, there were concerns that ED treatment services lacked experience in adapting care for patients with ASC, with one carer describing ED treatment as designed “for neurotypicals … it doesn’t take into account the autism” (Participant 8). Consequently, carers described having to take an active role in advocating for their child to receive appropriate care:

“I don’t know if [the autism diagnosis] is in progress or whether he’s been referred to anyone, or whatever … they forwarded it to the local Eating Disorder clinic who said “it’s got nothing to do with us” and they forwarded it to a central point who never got back to us or anything. I need to make a phone call to see if they’re going to do anything about it.” (Participant 1).

This participant’s experience reflects a common issue described by carers, which was that due to the severity of their loved one’s needs, and a lack of appropriate support by healthcare services, carers felt like they compensated by playing a more active role in their child’s treatment and increasing their caring responsibilities. A number of carers described how caring for their child increasingly dominated their lives as the ED progressed:

“We were supposed to be minding her, making sure that she wasn’t losing more weight, making sure that she was eating, basically me more than [husband] because he was going out to work every day. I had taken early retirement, I was very young, … forty-five. So I took early retirement from my job and education to mind [x]” (Participant 3).

As well as a perceived lack of appropriate support for their children, carers highlighted that they also felt that they could have benefited from adapted support for themselves and their families. Two carers described feeling like existing family-based treatments were not appropriate for people with autism, specifying difficulties with capacity for abstract thought and demand avoidance. This lack of support appeared to be related to increasing carer stress and exhaustion, with one carer describing how she feels that she cannot “give [her child] the best I can” as she is having to provide “24/7” care (Participant 10).

### Theme 3: impact on carers

Themes 2 and 3 were found to be closely interlinked in the analysis: the additional needs associated with ASC and EDs, and a lack of appropriate service provision, were perceived by carers as creating a significant burden of care, and dominating their lives and their identities: “it’s all-consuming, it doesn’t leave you time to think about much else” (Participant 8). For some carers, this represented a financial impact, including giving up their job to care for their child, or seeking private treatment. One carer felt that adapting their lives around the ED in this way in fact reinforced the ED itself:

“We are ingraining his problems. We have given him what he wanted. It’s like scraping and scraping a piece of wood. We have fed and been a martyr to his problems” (Participant 1).

In particular, the co-occurring ASC and ED was perceived as negatively impacting the family environment. Carers in relationships described how caring for their loved one put a strain on their relationship with their partner, particularly where the couple disagreed about how best to care for the child. The time and effort required to care for their child with autism and ED was also described as affecting their ability to support their other children, creating feelings of resentment and anger within the family:

“The younger girls suffered greatly at the head of all of this. I think [sibling’s name], because she was older, she was starting college, she was sort of moved on but she was affected by it as well. I think that they were resentful for a long time. Especially the youngest girl - she’s just turned twenty-four. I think she felt that [child with autism and ED] took me away from her” (Participant 3).

There was a sense that some carers were expending all their energy into caring for their child with ASC and EDs, at the expense of time to socialise, their career, and family relationships, resulting in isolation and their lives becoming centred around their caring role. One parent directly linked these difficulties to a lack of carer-orientated support from services:

“Parents do feel very alone and I do blame a little bit some of the hospitals and the staff … I think there needs to be more inclusion of parents” (Participant 2).

Therefore, caring for someone with co-occurring ASC and ED was described as extremely challenging by carers in this study, and was associated with significant personal sacrifice. There was a strong feeling throughout the interviews of hopelessness that appeared to be closely related to previous negative experiences accessing ineffective or un-adapted treatments, and their child’s lack of progress in recovery and continuing ED. One carer had the following response when asked “what would make your journey easier?” by the interviewer:

“To see progress. As a carer, you look at it and it’s frustrating, it dominates your life. You don’t really have a life – you feel like you’re sacrificing everything. And to be positive about that, you’ve got to see light at the end of the tunnel.” (Participant 1).

### Theme 4: supporting the supporters

Carers made a number of recommendations for changes which they felt would help support them in their role as carer. These broadly fell into two categories: improved treatment for their loved ones, which would help relieve the burden of care, and direct support and interventions designed to help the carers themselves. Improved treatments for their loved ones primarily focused around adapting ED treatments for needs associated with ASC, including training in ASC for ED clinicians, more personalised approaches and accommodating sensory needs in meal plans, including help for carers with meal support in their homes and a 24/7 helpline for autism/ED carers. There was a perception that people with ASC may benefit from more intensive care, including longer periods in treatment and the provision of day or inpatient services. In particular, there was a recognition by carers of adult children that some people with ASC require long term care, and a desire for additional support with the lifetime needs associated with this condition. For example, one carer described how their adult son remained dependent on them, and how they would benefit from support around helping him become more independent. Again, this was perceived as benefiting both the child and the carer:

“The last year has been really hard and he’s so regressed, just relies on me constantly. I wanted for him his own living space, but I want it also for me. It puts a strain on the relationship.” (Participant 7).

Carers also felt that they could benefit from support for themselves from services. In particular, there was an interest from carers in the provision of peer support groups, and support from others with lived experience of caring for someone either with an ED only, or both ASC and an ED. One carer described how she set up her own group in the absence of appropriate support, and felt that ED services could have done more to help with this. In response to the question “what would improvement look like”, she stated:

“Much, much more collaboration with people with lived experience. Because I’m that sort of person I felt like I needed to do something, I set up a parent/carer support group. Ideally it wouldn’t have been run by me at that point, because [child’s name] was even more unwell than she is now. But there wasn’t anything locally, so I felt with my skills and resources that was something I could set up, which I did. It didn’t get a very good take-up, but the eating disorder community team didn’t promote it.” (Participant 6).

Other support options suggested by carers including psychoeducation around co-occurring ASC and EDs, and services signposting additional sources of help. They also described the importance of feeling included in the treatment process, including more communication from clinicians. One carer felt that he would have benefited from clinicians acknowledging the difficulties associated with this comorbidity early in the process:

“I think local services could give a very honest picture and say “look, this is going to be really hard. This is going to stretch you and be really hard because of the nature of what it is, and particularly with autism, this is going to be challenging” … for me it would have been a help if someone said: “this is what you’re likely to experience”. (Participant 5).

### Theme 5: coronavirus

Seven out of the 11 participants (63,6%) who were interviewed mentioned the impact of the coronavirus pandemic on their lives and the people they were caring for. Negative impacts included their loved ones’ treatments being suspended, disrupted or moved online, heightening carer responsibilities in helping with their child’s treatment and recovery. Carers also reported that it was particularly difficult to obtain preferred foods and brands in shops due to stock shortages, and that both they and their child were experiencing worsening social isolation. For one carer, lockdown was the most difficult period she had experienced in caring for her child. The pressures of lockdown made it even harder for her to balance her caring role with her other responsibilities:

“So far these last few weeks have been the hardest, because of the pressure of - lockdown happening at the same time has made it more difficult, with the children and balancing things [managing person with autism and ED’s hospital admission]”. (Participant 8).

There was a suggestion that people with ASC and EDs may have particular difficulties around lockdown. For example, one parent described how their son struggled to be flexible around certain foods not being available in supermarkets due to his rigid thinking style: “he is unable to process that because it is not in his logic” (Participant 2).

A number of carers noted that, in the context of previous restrictions on their lives due to their caring role, their experience of lockdown was not significantly different compared to their everyday lives:

Carers coped by taking things ‘day by day; “we have had really bad days and we have had better days’” (Participant 2).

Being used to leading restricted lives meant that some carers felt that they had built up resilience from their caregiving experiences. Despite the fact that participants were recruited from two different sources, and that only those who were recruited from the PEACE Pathway had had their loved ones diagnosis confirmed in their clinical notes, participants across the sample reported remarkably similar stories.

## Discussion

This study aimed to explore the support needs and recommendations of carers of loved ones with co-occurring autism/ASC and EDs. As data collection took place during the initial UK coronavirus pandemic lockdown (March–July 2020), the analysis also captured the impact lockdown had on this population. The findings suggest that caring for an individual with both ASC and EDs has a significant impact on carers, which is exacerbated by the current lack of specialised or adapted services identified by previous research in this area [34]. In this context, carers made a number of recommendations for support options for this population, including peer-based support and psychoeducation. With the current paper replicating similar findings in prior studies evaluating needs associated with co-occurring autism and ED, this study adds strong support to arguments that associated populations, including patients, carers and clinicians, do require specialised adaptations and tailored treatment [21, 22, 34, 37].

Previous research on carers of loved ones with EDs has similarly described high levels of distress due to unmet needs, and a perceived lack of carer-focused support from clinical services [6, 7]. However, the current paper raises the possibility that carers of people with co-occurring ASC and EDs may experience additional difficulties or needs, compared to those caring for people with EDs only. Also, that existing ED services may not offer adapted treatment for patients with co-occurring ASC and related additional needs, which has been explored in previous research. The findings of this paper indicate that adaptations may also be required to enable better support for the carers of people with EDs and autism and their families [22, 34, 37]. The perceived lack of appropriate support by services for their children resulted in participants in this study making significant personal sacrifices in order to provide their loved ones with necessary care. This included carers giving up their careers and continuing to look after their offspring into middle age and later years. This is consistent with previous research on carers of children with autism: unlike EDs, which are mental illnesses with the possibility of recovery, autism is a lifelong condition. People with ASC may have lifelong support needs and so remain dependent on their families for longer periods [29, 33]. Therefore, it is possible that carers of people with ASC and EDs may have longer term support needs compared to carers of people with EDs only. Existing interventions for carers of people with EDs tend to be focused and time-limited, such as psychoeducational or skills-based workshops, and their benefits may diminish over time [13–15]. Further research should consider what kind of support options are most appropriate for this population, including the possibility of longer-term approaches. Existing ED carer or family interventions may also require adaptation to reflect specific needs associated with autism. For example, interventions designed to encourage family support around mealtime for people with AN could be adapted to consider how best to support individuals with sensory difficulties, or strong rituals around food and eating [3].

One idea raised in the current study was, rather than focusing on clinical service-provided interventions for carers, peer-based support for carers could be particularly beneficial for this population. Previous studies exploring peer support and peer mentoring in carer and ED fields have found that this approach lessens feelings of isolation and exclusion due to the sense of shared experience, and can create feelings of hope [40, 41]. With carers in the present study describing experiences of social isolation, hopelessness, and frustration with existing services due to a perceived lack of understanding of co-occurring ASC and ED, it is possible that peer-based support could be appropriate for this population. However, drawbacks described in previous studies include the difficulty of ending relationships at the end of the mentorship programme, and managing boundaries [41]. In addition, peer support alone may not be sufficient in helping carers, and additional or alternative sources of help may be required [40]. Therefore, the possibility of peer-based support options for carers of people with both ASC and EDs requires further research, including how clinicians and services can best support this process.

An additional, but originally unintended, aspect of caring for an individual with ASC and ED captured in the present study was the impact of coronavirus. The coronavirus pandemic has presented a number of challenges to people with EDs, their carers and their clinicians, including difficulty or changes in delivering treatment, a lack of access to preferred food, and social isolation [42–44]. One paper examining the experiences of carers highlighted that reduced professional support may have resulted in carers taking on an increased role in supporting their loved one, and managing their recovery [42]. Carer experiences in this study were consistent with these previous research suggestions, and one carer raised the possibility that these changes may be particularly difficult for people with ASC due to rigid thinking patterns and a lack of flexibility. The possibility that people with ASC and EDs may require additional support from ED services in adapting to the pandemic should be further explored.

A key strength of this study was its use of a peer-interviewing methodology. There is an increasing emphasis in the autism field on using participatory approaches to ensure that research has a positive impact on the ASC community [45]. Additionally, despite the slightly different methodology, the current study echoes previous research findings in this area, indicating that the needs and difficulties described in this paper are consistent across different samples [21, 22, 34, 37]. Secondly, previous qualitative research in this area has been almost exclusively female-focused [21, 22, 34]. A benefit of the current study was that 27% of participants were fathers, and 27% of the loved ones were male. Including male perspectives is important as although the majority of people with ASC are male, co-occurring autism and EDs in men are under-researched [46].

However, this study also has a number of limitations. Firstly, the diagnoses of the participants recruited from outside the SLAM ED [47] service were self-reported only and not further validated by the research team. This precludes the closer examination of the overlap between avoidant/restricted food intake disorder and ASC. Autism is a highly heterogenous condition: a more in-depth diagnostic assessment could have helped give a clearer picture of the exact needs and difficulties experienced by the loved ones in this study, informing the qualitative responses given by carers. Finally, whilst the use of qualitative methods gives insight into possible support options for this population, further research is required to empirically evaluate adapted interventions for carers of people with ASC and EDs.

## Conclusions

The findings of this study suggest that carers of people with co-occurring ASC and EDs may experience additional needs and difficulties compared to those caring for people with EDs only, and so require specific support. Recommendations for possible ways to support this population are outlined, but require further research.

## Data Availability

The datasets generated and/or analysed during the current study are not publicly available as participants did not consent for their data to be shared outside the author research group.

## Abbreviations

ASC: Autism Spectrum Condition
AN: Anorexia Nervosa
EDs: Eating Disorders
NHS: National Health Service
SLAM: South London and Maudsley NHS Foundation Trust
PEACE: Pathway for Eating disorders and Autism developed from Clinical Experience
